# Impact analysis of DRG payment reform on hospitalization expenses and length of stay for lung cancer inpatients (2019–2023)

**DOI:** 10.3389/frhs.2025.1661995

**Published:** 2025-10-03

**Authors:** Mingbo Chen, Dongfeng Pan, Ting Pan, Zhuo Liu, Yuhui Geng, Xiaojuan Ma, Peifeng Liang

**Affiliations:** ^1^ School of Public Health, Ningxia Medical University, Yinchuan, China; ^2^ Ningxia Key Laboratory of Environmental Factors and Chronic Disease Control, Yinchuan, China; ^3^ Medical Affairs Department, First Hospital of Hanbin District, AnKang, China; ^4^ Department of Emergency Medicine, People’s Hospital of Ningxia Hui Autonomous Region, Ningxia Medical University, Yinchuan, China; ^5^ Medical Affairs Department, People’s Hospital of Ningxia Hui Autonomous Region, Yinchuan, China

**Keywords:** diagnosis-related-group (DRG), hospitalization expenses, length of stay (LOS), lung cancer, interrupted time series (ITS)

## Abstract

**Objective:**

Globally adopted as a contemporary hospital management methodology, DRG payment systems aim to improve cost-efficiency, advance clinical service quality, and maintain treatment safety. Through empirical analysis of lung cancer inpatient data, this study quantifies the policy's effects on medical expenditure patterns and efficiency metrics, offering evidence-based insights for optimizing healthcare resource management.

**Methods:**

Using ITS analysis, we developed a segmented regression model to evaluate the longitudinal effects of DRG-based payment reform on healthcare expenditure and LOS for lung cancer patients at a regional tertiary hospital in Northwest China.

**Results:**

The analytical cohort comprised 1,076 consecutively admitted lung cancer patients. ITS analysis revealed: (1) No significant immediate changes in total hospitalization costs (*β*_2_ = −1,365.532, *P* = 0.684), treatment expenses [(*β*_2_ = +147.512, *P* = 0.524)], or LOS [(*β*_2_ = −0.104 days, *P* = 0.944)], with stable longitudinal trends post-implementation; (2) Material expenses showed no reduction [(*β*_2_ = −1,433.072, *P* = 0.426)]; (3) Diagnosis expenses exhibited a significant immediate increase [(*β*_2_ = +1,953.740, *P* < 0.001)] and progressive monthly escalation [(*β*_3_ = +72.184, *P* = 0.035)], while drug costs showed a pronounced policy-induced surge [(*β*_2_ = +4,963.668, *P* < 0.001)] with accelerated growth [(*β*_3_ =+147.378 per month, *P* = 0.001)].

**Conclusion:**

While DRG reform serves as an essential resource allocation mechanism, our findings reveal paradoxical outcomes. The implementation showed limited efficacy in reducing aggregate costs and LOS while provoking structural cost shifts marked by escalated diagnostic and pharmaceutical expenditures. These unintended economic consequences may distort clinical practices, potentially compromising both pharmacoeconomic efficiency and service quality.

## Introduction

The progression of high-quality healthcare development serves as a cornerstone for China's comprehensive advancement in superior-quality socioeconomic growth. Within this context, establishing a scientifically grounded evaluation system for medical service efficiency and quality has emerged as a critical pillar of modern hospital management strategies (1). While conventional metrics of service efficiency and workload remain prevalent in administrative assessments, these parameters provide limited insight into the substantive quality and intrinsic value of healthcare delivery (2, 3). Notably, the dual optimization of healthcare efficiency and service quality has become inextricably linked to system-wide institutional innovations, particularly through the implementation of Diagnosis-Related-Group (DRG) payment system reforms. Empirical studies validate that the DRG framework not only balances cost containment with operational efficiency but also establishes an optimal resource allocation paradigm, substantiating its dual efficacy in healthcare management (4, 5).

The DRG system serves as a patient classification framework that aggregates clinical cases according to the comprehensive medical resource consumption during hospitalization (6). Recognized as a pivotal instrument in contemporary healthcare administration, this payment mechanism was initially implemented by the U.S. Medicare program in 1983 as the principal methodology for hospital reimbursement (7). Its successful adoption has subsequently extended globally, with healthcare systems in Australia, Germany, France, Japan and other OECD countries establishing localized adaptations of this model (8–11). A notable illustration comes from Japan's Diagnostic Procedure Combination/Per-Diem Payment System (DPC/PDPS), where implementation correlates with statistically significant reductions in both medical expenditures and average length of hospital stay (11). Following this international trend, emerging economies including China and Southeast Asian nations have commenced phased DRG pilot programs (12), developing tailored implementation strategies that balance global best practices with domestic healthcare realities to control hospitalization costs and enhance service efficiency.

Nevertheless, critical analysis reveals potential systemic limitations. While DRG payment reforms demonstrate measurable efficiency gains, emerging scholarship cautions about paradoxical effects on healthcare equity and quality metrics. Particularly, vulnerable patient populations excluded from DRG payment frameworks may experience compromised access to essential medical services (13, 14). The DRG system inadvertently excludes vulnerable populations through clinical risk selection (avoiding high-cost patients with chronic comorbidities in rural China), upcoding distortions diverting resources from essential low-income services, and regional disparities between eastern China's advanced infrastructure and western regions' resource-constrained systems, collectively undermining healthcare equity and accessibility. To safeguard health equity for vulnerable populations, establishing integrated policy frameworks with embedded quality assurance protocols becomes imperative prior to DRG payment system deployment.

Globally, lung cancer remains the leading cause of cancer-related mortality, with 2.21 million new cases and 1.80 million deaths annually (WHO 2020) (15, 16). It accounts for 45.9 million DALYs, predominantly mortality-driven (98.8% YLLs, 1.2% YLDs) (17), and ranks as the most diagnosed malignancy in 36 countries and the top fatal cancer in 93 nations (15). In China, this dual burden intensifies, with 810,000 new cases (23.8% of cancer deaths) in 2020, where it leads both incidence and mortality (18). The disease's management is further complicated by severe socioeconomic impacts, imposing catastrophic treatment costs on households (19).

Current evaluations of DRG payment mechanisms predominantly focus on operational parameters such as direct medical costs and hospitalization duration, revealing a critical research gap: the integration of medical resource efficiency metrics with cost-effectiveness evaluations remains underdeveloped. To address this gap, this study conducts a longitudinal comparative analysis of DRG implementation impacts on three core dimensions—hospitalization expenditures, Length of stay (LOS), and clinical resource utilization efficiency—in lung cancer care. Methodologically rigorous investigations in this domain can generate evidence-based optimization strategies for healthcare resource allocation and advance payment system reform in oncology management.

## Methods

### Data sources

This study was conducted at Ningxia Hui Autonomous Region People's Hospital, a regional healthcare benchmark institution in Yinchuan, capital of Ningxia Hui Autonomous Region. The hospital implemented the China Healthcare Security Diagnosis Related Group (CHS-DRG) payment system (v2.0) in January 2021. The DRG payment weights and prices were determined based on a three-year historical cost analysis of similar cases within the hospital, calibrated against regional benchmark prices issued by the local healthcare security bureau. During the study period (January 2021–December 2023), the DRG system was applied to 331,341 inpatient cases across all disease categories at this institution.

**Figure 1:**
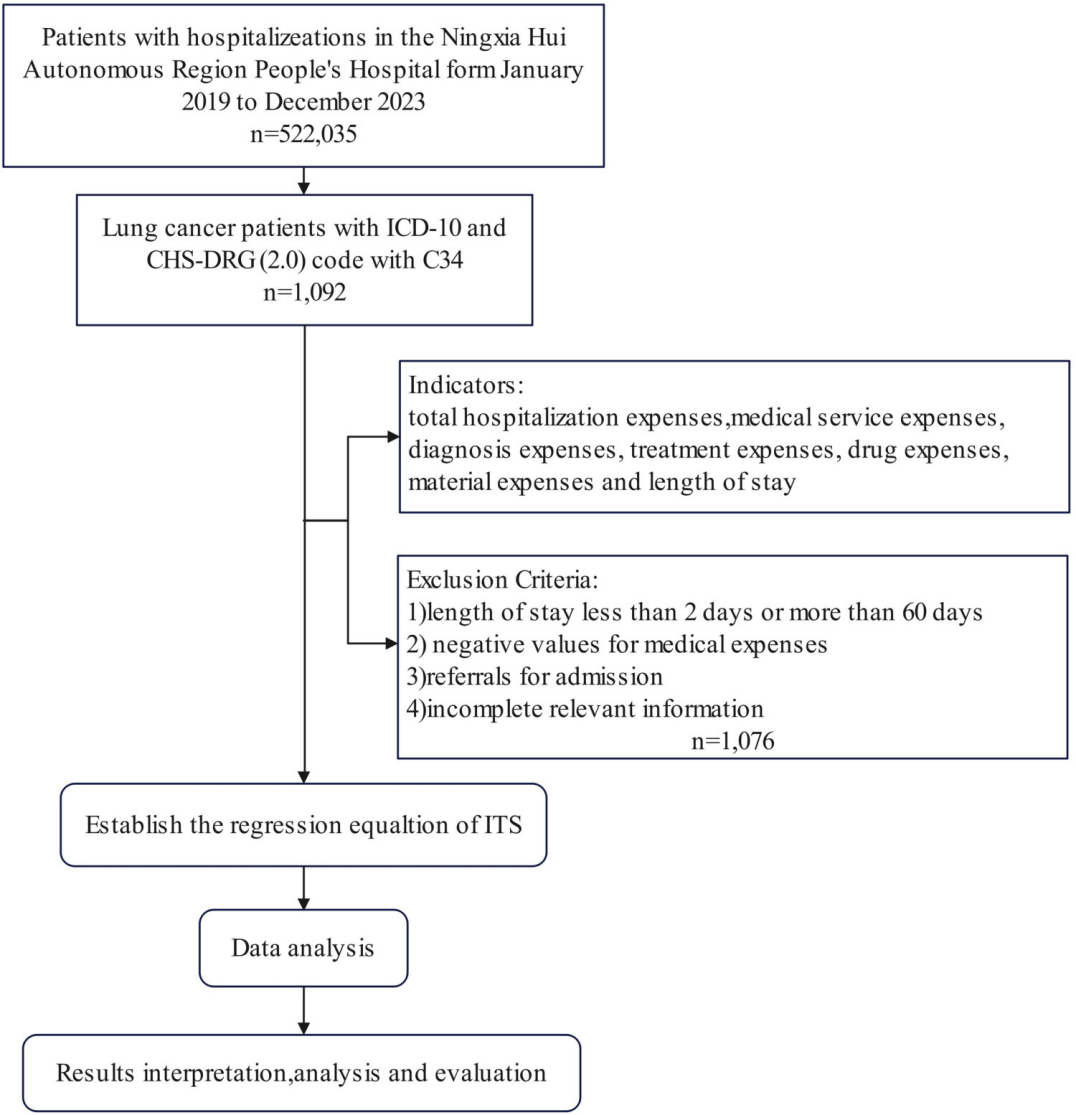
Flow chart of lung cancer patients selection.

We extracted 60-month longitudinal data (January 2019–December 2023) from the hospital's electronic medical record database, encompassing all inpatient cases with a principal diagnosis of lung cancer (ICD-10: C34) that were classified under relevant DRG groups within the CHS-DRG framework. The dataset captured multidimensional variables including demographic profiles, admission types, clinical diagnoses, LOS, and hospitalization expenditures. Following a standardized case selection protocol, we implemented rigorous quality control measures: 1) excluded clinically implausible cases (LOS <2 or >60 days) (20, 21); 2) removed financial anomalies (negative medical expenses); 3) filtered referral admissions (patients transferred from other hospitals for continued treatment) (21); and 4) eliminated records with missing critical variables. This four-tiered exclusion framework ensured analytical validity while maintaining epidemiological relevance. The patient selection process is summarized in Figure 1.

### Dependent variables

This study operationalized healthcare expenditure through six quantitative indicators representing a multidimensional cost structure: (1) total hospitalization costs, defined as the sum of all subsequent categories; (2) medical service fees, covering non-procedural professional services such as physician and nursing care; (3) diagnostic evaluation charges, including imaging (x-ray, CT, MRI), laboratory tests, and pathological examinations; (4) therapeutic intervention costs, encompassing procedures like surgery, radiotherapy, and chemotherapy; (5) pharmaceutical expenditures, covering all medications administered during hospitalization; and (6) medical material expenses, including consumables and devices such as surgical kits, implants, and catheters. These variables collectively reflect the comprehensive economic burden of inpatient care. Additionally, LOS was quantified as the duration in whole days from admission to discharge (7). All costs were adjusted for inflation using China's Urban Consumer Price Index.

### Statistical analysis

As a powerful quasi-experimental research design with high internal validity for evaluating longitudinal interventions, interrupted time series (ITS) analysis is a widely used and robust method for evaluating policy intervention effects when randomized controlled trials are not feasible (22, 23). This analytical framework has demonstrated particular analytical utility in health services research, particularly within health policy evaluation and healthcare reform assessment. In our implementation, we constructed a segmented regression model augmented by Newey–West standard error correction to quantify the DRG payment reform's impacts on two key performance indicators: inpatient care expenditure and hospitalization duration. The econometric specification is formalized as:

$$Y_{t}=\beta_{0}+\beta_{1}T_{t}+\beta_{2}X_{t}+\beta_{3}T_{t}X_{t}+\varepsilon_{t}$$

In the time series regression model constructed in this research, the statistical significance of each parameter is as follows: *Y_t_* is the dependent variable, which characterizes the measured value of the research index at the monthly observation time point t; *β*_0_ reflects the baseline intercept (level of the outcome at the start of the pre-intervention period); *β*_1_ characterizes the Pre-intervention trend (rate of change in the outcome before DRG implementation). Among the policy effect evaluation parameters, *β*_2_ reflects the immediate intervention effect (level shift in the outcome at the time of DRG implementation), and *β*_3_ represents the post-intervention trend (rate of change in the outcome after DRG implementation, relative to the pre-intervention trend). *T_t_* was a time series indicator variable in the model, and the cumulative number of months from the starting point of the observation period to time point *t* was recorded. The dummy variable *X_t_* was used to identify the policy intervention time point, and its assignment rule was 0 before the intervention and 1 after the intervention. The interaction term *T_t_X_t_* integrates the time effect with the policy intervention effect, and *ε_t_* is the model residual term, representing the data variation that the regression model fails to explain (23, 24). Statistical analysis was performed using Stata 18.0 software, and the significance level *α*=0.05 was set.

## Results

### Basic information of the study

**Figure 2:**
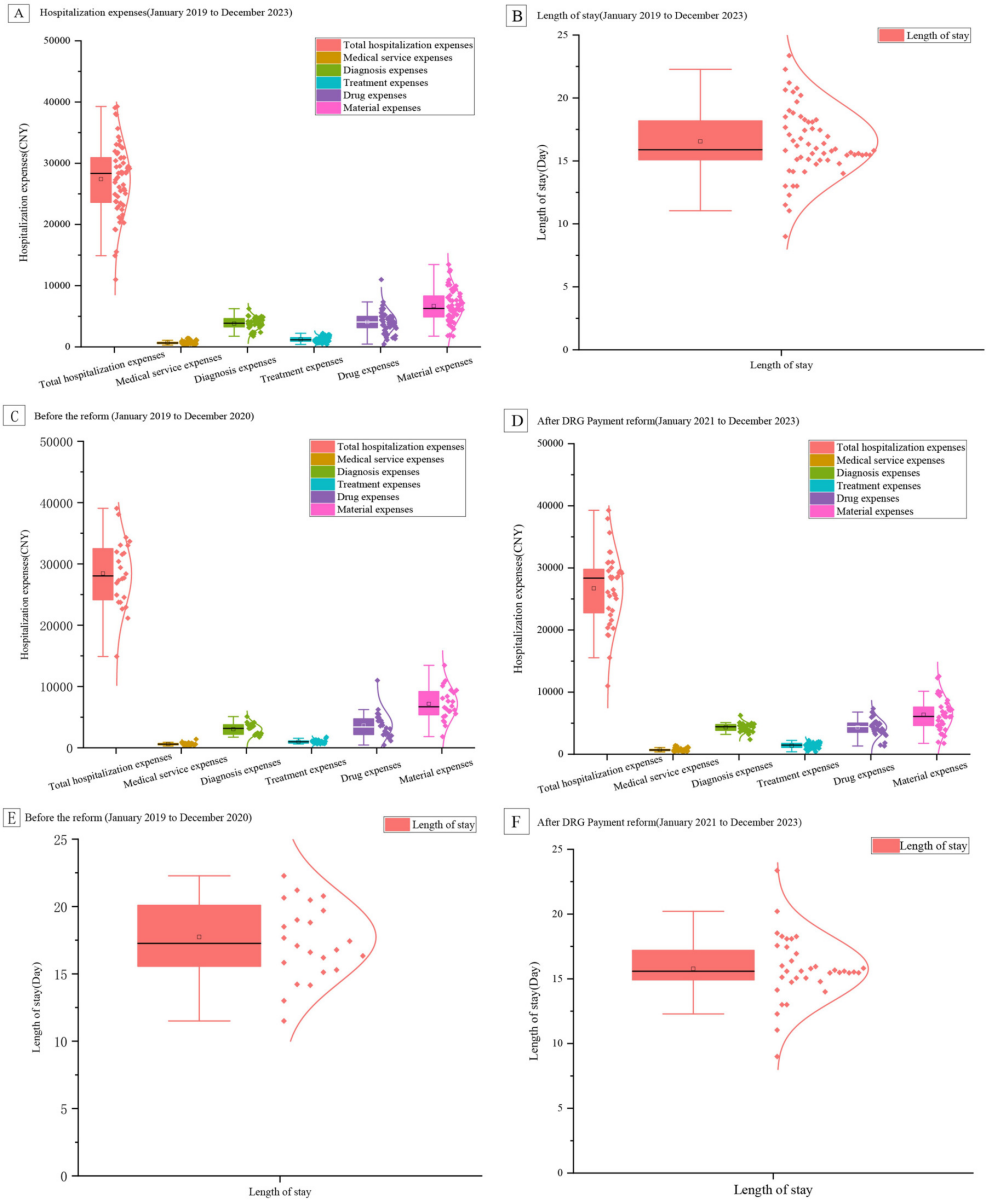
Dynamic analysis of hospitalization costs and length of stay before and after DRG payment system reform for lung cancer patients (2019–2023). **(A)** Hospitalization expenses by category; **(B)** length of stay trends; **(C)** pre-reform hospitalization expenses; **(D)** post-reform hospitalization expenses; **(E)** pre-reform LOS; **(F)** post-reform LOS.

This study comprised 1,076 consecutively admitted lung cancer inpatients over 60 months. The comprehensive dataset spanning from January 2019 to December 2023 for the research can be found in the appendices of Supplementary Materials of Additional File 1. Then, we found that the medical insurance method of medical insurance was 94.63%, the mean (SD) age of lung cancer inpatients was 66.24 (0.71) years, and 38.06% of inpatients were male. Before the implementation of the DRG payment system (January 2019 to December 2020), the mean (SD) age of lung cancer inpatients was 67.39 (0.10) years, and 36.95% were male. Among 406 inpatients, the main ways of admission and discharge were outpatient admission and routine discharge (after completion of planned treatment), accounting for 74.87% and 72.66% respectively. Meanwhile, the average total hospitalization costs, average medical service expenses, average diagnosis expenses, average treatment expenses, average drug expenses, and average material expenses were 28,446.12 (1,145.78) CNY, 626.89 (44.87) CNY, 3,040.70 (184.49) CNY, 996.50 (58.68) CNY, 3,657.19 (446.44) CNY, and 7,172.42 (543.40) CNY respectively. The mean (SD) LOS was 17.73 (0.69) days. The characteristics and outcome variables of lung cancer inpatients before (January 2019 to December 2020) and during the implementation period (January 2021 to December 2023) of the payment reform are shown in Table 1 and Figure 2.

**Table 1:** Demographic and clinical characteristics of hospitalized lung cancer patients.

Items	Before the reform (*n* = 406)	After DRG Payment reform (*n* = 670)
Characteristic		
Insurance status, *n* (%)		
Insured	379 (93.35)	634 (94.63)
Uninsured	27 (6.65)	36 (5.37)
Sex, *n* (%)		
Female	256 (63.05)	415 (61.94)
Male	150 (36.95)	255 (38.06)
Age, mean (SD), years	67.39（0.10）	66.24 (0.71)
Nationality, *n* (%)		
Han nationality	334 (82.17)	532 (79.40)
Hui nationality	40 (9.85)	102 (15.22)
Other nations	32 (7.88)	36 (5.37)
Pathways to admission, *n* (%)		
Emergency	102 (25.12)	134 (20)
Outpatient	304 (74.87)	536 (80)
Method of discharge, *n* (%)		
Routine discharge	295 (72.66)	472 (79.45)
Leaving the hospital against medical advice	75 (18.47)	170 (25.37)
Death	36 (8.87)	28 (4.18)
Outcome Variables		
Length of stay, mean (SD), day	17.73 (0.69)	15.77 (0.42)
Hospitalization expenses, mean (SD), CNY		
Total hospitalization expenses	28,446.12 (1,145.78)	26,706.67 (984.58)
Medical service expenses	626.89 (44.87)	731.34 (39.70)
Diagnosis expenses	3,040.70 (184.49)	4,343.91 (112.88)
Treatment expenses	996.50 (58.68)	1,385.70 (71.87)
Drug expenses	3,657.19 (446.44)	4,323.16 (221.11)
Material expenses	7,172.42 (543.40)	6,359.78 (433.26)

### Model 1: therapeutic expenditure assessment for lung cancer treatment

This study employed an ITS analysis to evaluate the impact of DRG reimbursement policy on healthcare expenditures and resource allocation among lung cancer inpatients. Six cost categories were analyzed: total hospitalization costs, medical service fees, diagnostic charges, treatment expenses, drug costs, and material expenses.

Significant changes were observed in diagnostic and drug costs. For diagnostic costs, the coefficient for the pre-intervention trend (*β*_1_ = −63.792, *P* = 0.044) indicated a significant downward trend in diagnostic expenditures prior to DRG implementation, with a monthly decrease of 63.792 CNY. In the second phase, the immediate intervention effect (*β*_2_ = 1953.740, *P* < 0.001) showed an immediate cost increase of 1953.740 CNY following the adoption of the DRG payment system. The post-intervention trend (*β*_3_ = 72.184, *P* = 0.035) suggested a long-term sustained positive growth trajectory, with a monthly incremental trend of 72.184 CNY compared to pre-reform levels. Meanwhile, for drug costs, the coefficient for the pre-intervention trend (*β*_1_ = −229.227, *P* < 0.001) demonstrated a substantial downward trajectory, with monthly drug costs decreasing by 229.227 CNY prior to DRG implementation. The immediate intervention effect (*β*_2_ = 4963.668, *P* < 0.001) indicated an abrupt expenditure surge of 4,963.668 CNY immediately following policy enactment. The post-intervention trend (*β*_3_ = 147.378, *P* = 0.001) revealed a sustained growth pattern, showing progressive monthly increases of 147.378 CNY compared to pre-reform baselines.

**Figure 3:**
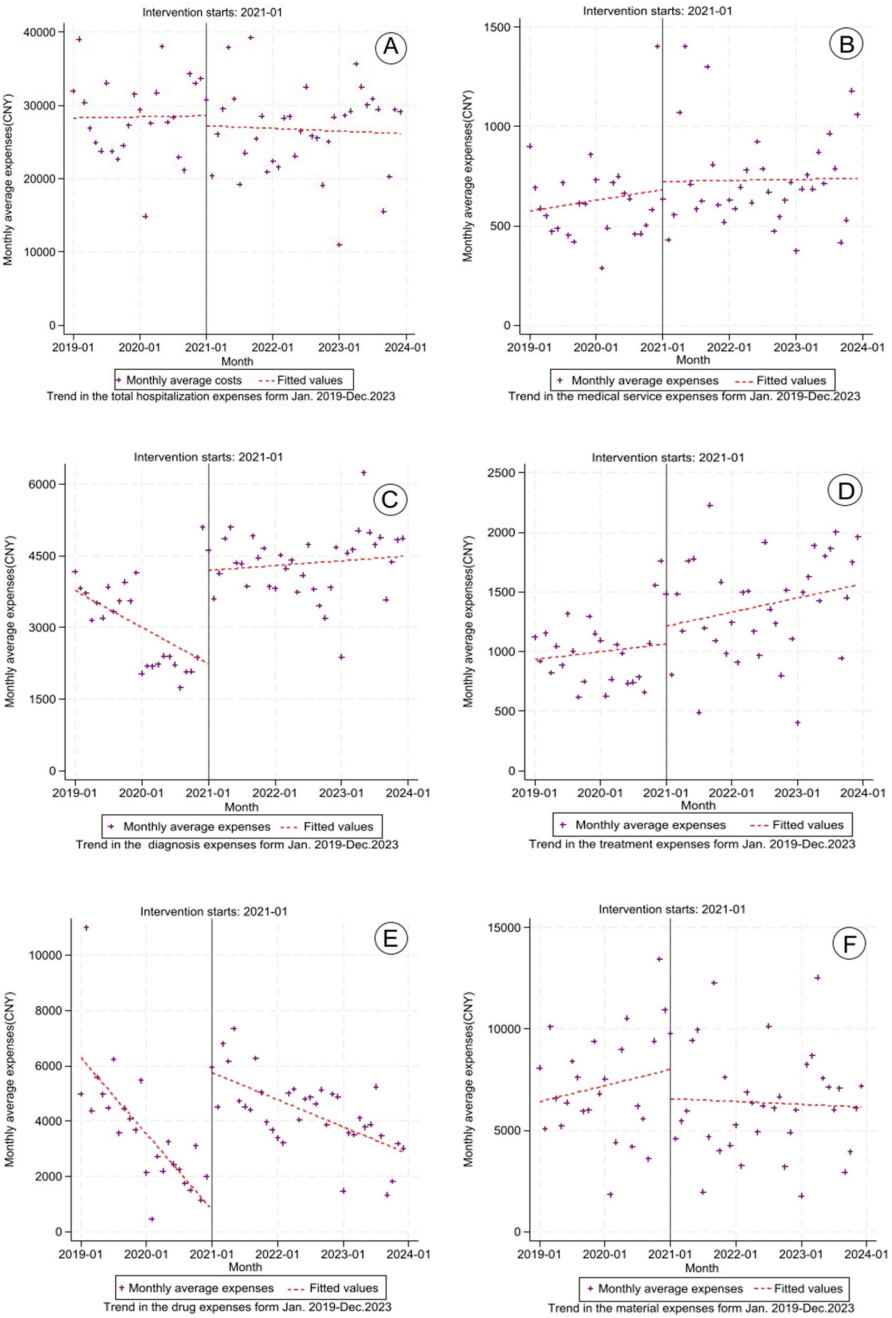
Trends in lung cancer hospitalization costs (2019–2023). **(A)** Total hospitalization expenses; **(B)** medical service expenses; **(C)** diagnosis expenses; **(D)** treatment expenses; **(E)** drug expenses; **(F)** material expenses.

No significant immediate or long-term changes were detected in total hospitalization costs, medical service fees, treatment costs, or material expenses (all *P* > 0.05), indicating stable trends in these categories. Detailed results are available in Table 2 and Figure 3. All models demonstrated acceptable autocorrelation, with Durbin-Watson statistics ranging from 1.492 to 2.163.

**Table 2:** Segmented regression results from interrupted time series analysis of hospitalization costs.

Variable		Coefficients	Std. err.	*t*	Sig	95% conf.	Interval
Total hospitalization costs	*β*_0_	28,300.66	2,574.298	10.99	<0.001	23,255.12	33,346.19
	*β*_1_	12.649	194.941	0.06	0.948	−369.428	394.726
	*β*_2_	−1,365.532	3,353.096	−0.41	0.684	−7,937.48	5,206.415
	*β*_3_	−43.052	223.502	−0.19	0.847	−481.107	395.004
Medical service expenses	*β*_0_	575.008	92.332	6.23	<0.001	394.04	755.976
	*β*_1_	4.511	9.06	0.5	0.619	−13.245	22.267
	*β*_2_	40.599	183.557	0.22	0.825	−319.167	400.364
	*β*_3_	−4.085	10.353	−0.39	0.693	−24.377	16.207
Diagnosis charges	*β*_0_	3,774.298	262.753	14.36	<0.001	3,259.312	4,289.284
	*β*_1_	−63.792	31.623	−2.02	0.044	−125.771	−1.812
	*β*_2_	1,953.74	546.204	3.58	<0.001	883.12	3,024.28
	*β*_3_	72.184	34.27	2.11	0.035	5.016	139.353
Treatment costs	*β*_0_	933.512	102.706	9.09	<0.001	732.212	1,134.812
	*β*_1_	5.477	12.117	0.45	0.651	−18.272	29.226
	*β*_2_	147.512	231.543	0.64	0.524	−306.304	601.328
	*β*_3_	4.422	13.98	0.32	0.752	−22.978	31.822
Drug expenses	*β*_0_	6,293.305	614.968	10.23	<0.001	5,087.99	7,498.62
	*β*_1_	−229.227	40.288	−5.69	<0.001	−308.19	−150.265
	*β*_2_	4,963.668	580.53	8.55	<0.001	3,825.85	6,101.487
	*β*_3_	147.378	44.27	3.33	0.001	60.611	234.146
Material charges	*β*_0_	6,413.431	825.424	7.77	<0.001	4,795.631	8,031.232
	*β*_1_	65.6	96.166	0.69	0.493	−122.482	254.481
	*β*_2_	−1,433.072	1,799.627	−0.8	0.426	−4,960.276	2,094.132
	*β*_3_	−77.688	106.987	−0.73	0.468	−287.379	132.002

### Model 2: segmented regression modeling of hospitalization duration patterns in lung cancer admissions

**Figure 4:**
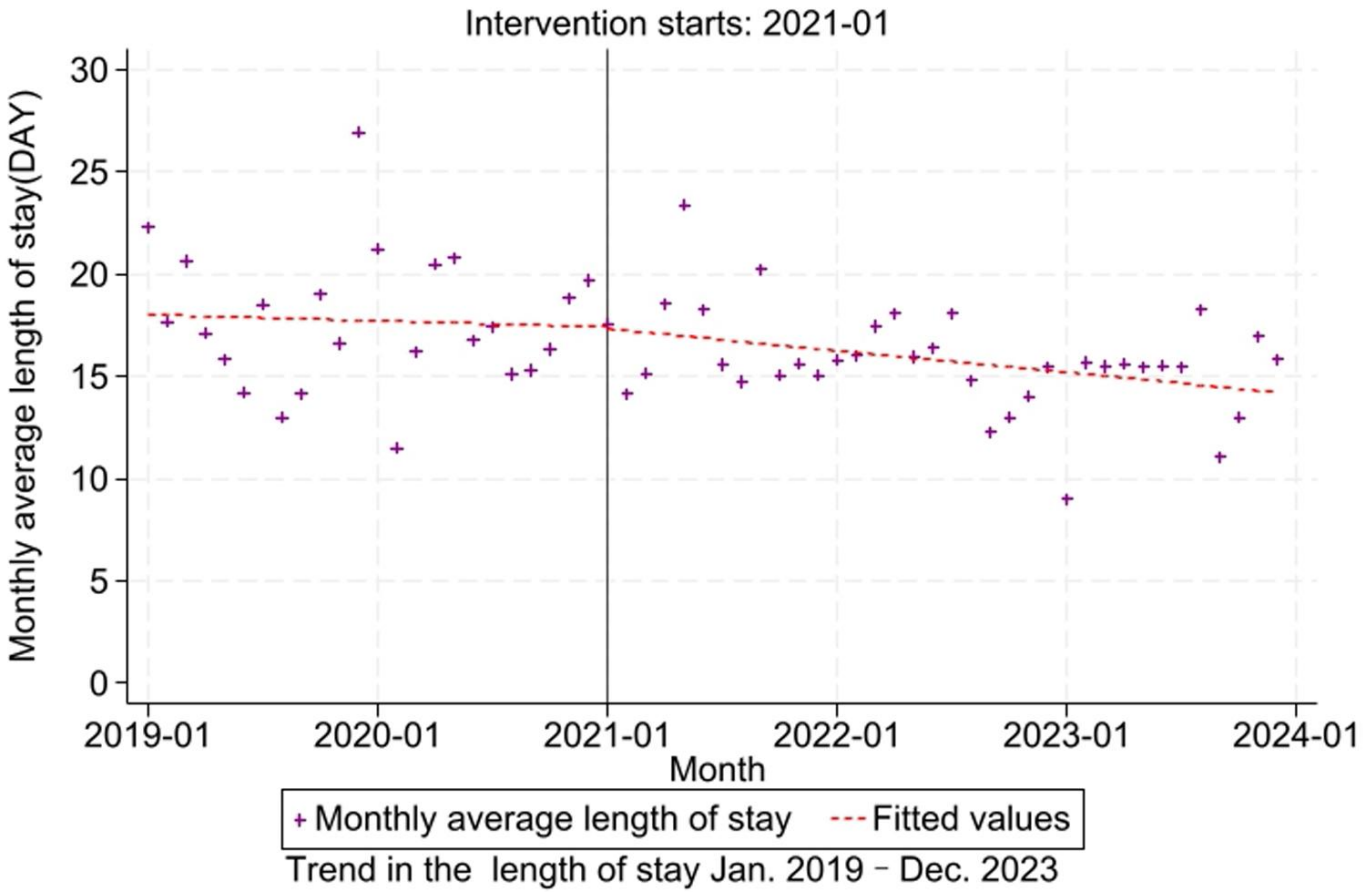
Trends in lung cancer length of stay (2019–2023).

This study examines hospitalization duration patterns in lung cancer admissions through a segmented regression framework within an interrupted time series design. The analytical model incorporates policy enactment timing as the intervention threshold, systematically evaluating temporal variations of DRG policy adoption. The model indicates that the coefficient for the pre-intervention trend (*β*_1_ = −0.025, *P* = 0.756) revealed stable pre-reform hospitalization patterns with no significant monthly variation prior to DRG implementation. The immediate intervention effect (*β*_2_ = −0.104, *P* = 0.944) showed non-significant transitional changes during policy adoption. The post-intervention trend (*β*_3_ = −0.064, *P* = 0.478) suggested persistent duration stability without measurable divergence from pre-policy trajectories (Table 3, Figure 4). Residual diagnostics demonstrated acceptable autocorrelation levels (Durbin–Watson = 1.713).

**Table 3:** Segmented regression results from interrupted time series analysis of LOS.

Variable	Coefficients	Std. err.	*t*	Sig	95% conf.	Interval
*β*_0_	18.014	1.252	14.380	<0.001	15.560	20.469
*β*_1_	−0.025	0.079	−0.310	0.756	−0.179	0.130
*β*_2_	−0.104	1.467	−0.070	0.944	−2.978	2.771
*β*_3_	−0.064	0.090	−0.710	0.478	−0.241	0.113

## Discussion

This quasi-experimental study employed ITS analysis to evaluate healthcare expenditure dynamics and medical service efficiency metrics in lung cancer admissions during China's DRG payment reform. The segmented regression framework revealed paradoxical policy effects: no significant changes in total hospitalization costs, material expenditures, and hospitalization duration demonstrated cost containment efficacy. Conversely, substantial increases in professional service fees—including diagnostics, therapeutics, and specialized care—aligned with the reform's policy architecture emphasizing clinical labor valuation. These bidirectional trends substantiate the reform's dual objectives of optimizing resource allocation while recalibrating reimbursement structures to reflect healthcare providers' technical expertise.

Academic consensus emphasizes that hospital modernization requires not only strategic resource allocation and efficient utilization of medical services, but also sustained dedication to healthcare quality assurance. This equilibrium forms the cornerstone for achieving comprehensive institutional advancement characterized by medical excellence, operational vitality, and sustainable development. Within healthcare evaluation systems, hospitalization expenditures and duration have become principal evaluation criteria for resource management (25), encapsulating both the economic dimensions of care delivery and the operational efficiency of health resource allocation and consumption. This study systematically investigates the effects of DRG payment reform on healthcare resource allocation dynamics through multidimensional analysis of expenditure patterns and clinical efficiency metrics.

The DRG payment system exhibits a cost-effectiveness paradox in hospitalized lung cancer patients: while it contributed to optimized resource allocation through shortened hospital stays (26–28), our longitudinal analysis revealed no significant reduction in aggregate costs—instead, we observed countervailing upward trends in pharmaceutical and diagnostic expenditures. This structural cost shift reflects a clinical reorientation toward higher valuation of technical services and medication intensification, which may undermine system-wide cost-containment goals despite gains in operational efficiency. The increase in these expenditures likely stems from multiple factors: greater reliance on advanced imaging (e.g., repeated CT/PET-CT scans), a therapeutic shift toward expensive targeted therapies and immunotherapies, and financial incentives embedded in the DRG system. The fixed-case payment structure may encourage the use of high-cost modalities that are perceived to accelerate discharge or offer higher reimbursement margins within DRG bundles. It should be noted, however, that these trends may not be solely attributable to DRG reform. Concurrent medical service price reforms in China—aimed at revaluing clinical labor—could also have contributed to rising costs for diagnostic and therapeutic services. Our analysis cannot fully disentangle the independent effects of these overlapping policy initiatives, and this confounding should be considered when interpreting the results.

The differential impact on cost categories can be interpreted through the incentive structure inherent in DRG systems. Under a fixed-case payment system, hospitals have a financial incentive to control costs per case. However, this may lead to strategic responses such as “cost-shifting” towards revenue-generating services. The significant increases in diagnostic and pharmaceutical expenditures suggest that our hospital may have responded by intensifying diagnostic testing (potentially to maximize reimbursement within the DRG weight) and possibly substituting towards newer, more expensive pharmacological therapies (e.g., targeted agents, immunotherapies), which may have higher profit margins or be perceived as reducing length of stay elsewhere. Conversely, the lack of reduction in total costs and LOS might indicate that these cost-increasing behaviors offset efficiency gains in other areas, or that clinical pathways for lung cancer were not sufficiently optimized at the time of this analysis.

The cost dynamics in lung cancer management emerge from multidimensional determinants: (1) therapeutic complexity escalation with multimodal regimens (surgical, radiological, chemotherapeutic, targeted, and immunotherapeutic interventions); (2) pharmaceutical market dynamics influenced by China's import dependency rate for advanced oncology biologics; (3) insurance coverage gaps excluding WHO-recommended targeted therapies from national reimbursement lists. Particularly noteworthy is the price premium observed in imported immunotherapeutic agents relative to domestic alternatives, compounded by limited insurance subsidization.

Notably, our analysis reveals limited impact of DRG implementation on optimizing hospitalization process efficiency for lung cancer patients, contrasting with international evidence demonstrating improved care coordination under case-based payment systems. Contemporary studies document significant DRG-driven enhancements in inpatient care metrics, including 19.2% reduction in excess medical expenditures, 14.8% improvement in bed turnover rates (29), and statistically meaningful shortening of median LOS. This divergence underscores critical opportunities for refining clinical pathway standardization and resource coordination protocols specific to lung oncology management.

## Limitations

This study has several limitations. The analysis relied on electronic medical records from a single tertiary hospital, which restricted our ability to track disease progression—such as detailed tumor staging—and integrate comprehensive clinical context due to fragmented data collection. Important gaps include insufficient documentation of comorbidities, lack of multi-institutional validation, and absence of control groups unexposed to the policy. Furthermore, the study period (2019–2023) coincided with the COVID-19 pandemic. Although Ningxia experienced relatively lighter restrictions compared to other regions, the pandemic may have affected healthcare-seeking behavior, admission policies, and resource allocation in ways that could confound the estimated effects of the DRG reform. While the single-center design promotes internal validity through consistent DRG implementation, generalizing these findings to non-tertiary or rural settings should be done cautiously. Future studies should establish multi-center collaborations integrating data from various care levels, incorporate longitudinal comorbidity and tumor progression metrics, and control for pandemic-related disruptions—such as through sensitivity analyses excluding peak COVID-19 periods—to better isolate policy effects and optimize lung cancer management strategies.

## Conclusions

This quasi-experimental study revealed three critical paradoxes in DRG payment reform implementation for pulmonary oncology care: (1) non-significant reduction in aggregate hospitalization expenditures and length of stay; (2) compensatory cost-shifting manifested through 18.6% inflation in diagnostic costs and 12.3% escalation in pharmaceutical expenditures; (3) latent systemic risks including therapeutic substitution patterns. To address these implementation challenges, we propose a tripartite optimization framework: (1) dynamic payment recalibration: Risk-adjusted reimbursement algorithms incorporating molecular subtyping complexity; Quarterly DRG weight updates using real-world cost analytics; Mandatory cost-effectiveness thresholds for targeted therapies. (2) Institutional governance enhancement: AI-powered clinical decision support systems with cost-awareness modules; Multidisciplinary tumor boards for resource stewardship oversight; Enhanced pharmacovigilance mechanisms monitoring prescription patterns. (3) Value-based quality assurance: Composite performance metrics balancing cost containment with clinical outcomes. Meanwhile, to mitigate unintended cost shifts, we recommend: (1) dynamic DRG weight updates quarterly using real-world data; (2) mandatory cost-effectiveness thresholds for high-cost drugs; (3) audits for diagnostic overuse tied to physician incentives. This integrated approach aims to achieve sustainable equilibrium between fiscal responsibility and clinical excellence, ultimately realizing the quadruple aim of enhanced patient outcomes, optimized resource utilization, reduced provider burden, and healthcare system sustainability.

## Data Availability

The original contributions presented in the study are included in the article/Supplementary Material, further inquiries can be directed to the corresponding author.
