# Supplementary material for: Impact analysis of DRG payment reform on hospitalization expenses and length of stay for lung cancer inpatients (2019–2023)
Source: Front Health Serv. 2025 Oct 3;5:1661995. doi: 10.3389/frhs.2025.1661995 (PMC12531130; doi:10.3389/frhs.2025.1661995)
Supplement: Supplementary file 1 [file Table1.doc]

Additional file 1: Changes in total hospitalization costs,medical service expenses,diagnostic fees,treatment costs,drug expenditures,material expenses, and length of stay for lung cancer patients in the People's Hospital of Ningxia Hui Autonomous Region of Yinchuan from January 2019 to December 2023.

| Date | Total hospitalization expenses  (CNY ) | Medical service expenses  (CNY ) | Diagnosis expenses (CNY ) | Treatment expenses  (CNY ) | Drug expenses  (CNY ) | Material expenses (CNY ) | Length of stay (DAY) | Time | Intervention | Time after intervention |
| --- | --- | --- | --- | --- | --- | --- | --- | --- | --- | --- |
| 19-Jan | 31965.52 | 900.86 | 4167.63 | 1121.65 | 4973.33 | 8075.42 | 22.28 | 1.00 | 0.00 | 0.00 |
| 19-Feb | 39068.07 | 692.50 | 3819.77 | 918.73 | 10995.54 | 5071.29 | 17.67 | 2.00 | 0.00 | 0.00 |
| 19-Mar | 30401.53 | 587.04 | 3720.32 | 1152.58 | 4384.29 | 10098.08 | 20.64 | 3.00 | 0.00 | 0.00 |
| 19-Apr | 26885.02 | 549.42 | 3152.93 | 821.52 | 5587.07 | 6582.29 | 17.09 | 4.00 | 0.00 | 0.00 |
| 19-May | 24926.97 | 472.59 | 3517.75 | 1043.60 | 4971.68 | 5212.35 | 15.83 | 5.00 | 0.00 | 0.00 |
| 19-Jun | 23753.44 | 486.88 | 3198.72 | 882.99 | 4472.61 | 6366.59 | 14.22 | 6.00 | 0.00 | 0.00 |
| 19-Jul | 33060.94 | 716.07 | 3852.80 | 1316.80 | 6237.95 | 8404.29 | 18.50 | 7.00 | 0.00 | 0.00 |
| 19-Aug | 23721.18 | 451.86 | 3330.95 | 1003.79 | 3560.75 | 7614.23 | 13.00 | 8.00 | 0.00 | 0.00 |
| 19-Sep | 22651.79 | 419.28 | 3550.40 | 617.61 | 4451.79 | 5938.29 | 14.16 | 9.00 | 0.00 | 0.00 |
| 19-Oct | 24541.61 | 613.13 | 3953.31 | 749.66 | 4091.85 | 5993.17 | 19.00 | 10.00 | 0.00 | 0.00 |
| 19-Nov | 27296.14 | 611.03 | 3549.72 | 1293.68 | 3673.95 | 9390.71 | 16.60 | 11.00 | 0.00 | 0.00 |
| 19-Dec | 31545.06 | 859.39 | 4145.95 | 1151.13 | 5473.47 | 6804.37 | 26.95 | 12.00 | 0.00 | 0.00 |
| 20-Jan | 29404.92 | 730.56 | 2034.58 | 1092.61 | 2125.76 | 7534.68 | 21.21 | 13.00 | 0.00 | 0.00 |
| 20-Feb | 14896.94 | 287.76 | 2196.08 | 628.50 | 456.00 | 1823.07 | 11.50 | 14.00 | 0.00 | 0.00 |
| 20-Mar | 27592.98 | 489.78 | 2185.40 | 767.29 | 2718.45 | 4405.18 | 16.20 | 15.00 | 0.00 | 0.00 |
| 20-Apr | 31753.40 | 717.32 | 2227.95 | 1058.36 | 2195.49 | 8978.09 | 20.48 | 16.00 | 0.00 | 0.00 |
| 20-May | 38072.69 | 748.64 | 2403.65 | 986.20 | 3238.24 | 10524.51 | 20.78 | 17.00 | 0.00 | 0.00 |
| 20-Jun | 27700.47 | 665.13 | 2391.16 | 735.63 | 2445.70 | 4199.90 | 16.78 | 18.00 | 0.00 | 0.00 |
| 20-Jul | 28377.59 | 637.14 | 2220.49 | 743.77 | 2256.26 | 6182.49 | 17.43 | 19.00 | 0.00 | 0.00 |
| 20-Aug | 22941.37 | 459.36 | 1740.87 | 785.85 | 1743.04 | 5560.22 | 15.12 | 20.00 | 0.00 | 0.00 |
| 20-Sep | 21154.57 | 461.20 | 2072.26 | 659.82 | 1498.98 | 3592.50 | 15.29 | 21.00 | 0.00 | 0.00 |
| 20-Oct | 34311.61 | 503.39 | 2076.12 | 1069.96 | 3100.24 | 9399.46 | 16.33 | 22.00 | 0.00 | 0.00 |
| 20-Nov | 33007.70 | 581.56 | 2368.14 | 1555.28 | 1138.02 | 13452.45 | 18.81 | 23.00 | 0.00 | 0.00 |
| 20-Dec | 33675.46 | 1403.37 | 5099.71 | 1758.87 | 1982.09 | 10934.51 | 19.70 | 24.00 | 0.00 | 0.00 |
| 21-Jan | 30828.87 | 634.42 | 4613.15 | 1484.28 | 5951.16 | 9776.02 | 17.57 | 25.00 | 1.00 | 25.00 |
| 21-Feb | 20354.10 | 428.67 | 3605.47 | 804.65 | 4512.35 | 4594.56 | 14.13 | 26.00 | 1.00 | 26.00 |
| 21-Mar | 26100.82 | 555.78 | 4136.91 | 1482.57 | 6796.59 | 5450.95 | 15.13 | 27.00 | 1.00 | 27.00 |
| 21-Apr | 29524.90 | 1070.76 | 4859.43 | 1173.20 | 6157.26 | 5961.34 | 18.53 | 28.00 | 1.00 | 28.00 |
| 21-May | 37933.56 | 1403.37 | 5099.71 | 1758.87 | 7342.88 | 9441.50 | 23.37 | 29.00 | 1.00 | 29.00 |
| 21-Jun | 30918.98 | 709.34 | 4350.32 | 1778.52 | 4721.95 | 9955.06 | 18.28 | 30.00 | 1.00 | 30.00 |
| 21-Jul | 19212.74 | 583.85 | 4340.02 | 489.08 | 4518.43 | 1957.23 | 15.58 | 31.00 | 1.00 | 31.00 |
| 21-Aug | 23500.15 | 625.14 | 3862.99 | 1196.12 | 4400.28 | 4668.73 | 14.74 | 32.00 | 1.00 | 32.00 |
| 21-Sep | 39278.15 | 1300.83 | 4912.97 | 2227.07 | 6266.67 | 12280.58 | 20.21 | 33.00 | 1.00 | 33.00 |
| 21-Oct | 25476.60 | 808.07 | 4459.75 | 1091.87 | 5040.64 | 3978.43 | 15.06 | 34.00 | 1.00 | 34.00 |
| 21-Nov | 28545.03 | 606.21 | 4657.87 | 1583.29 | 3965.84 | 7630.31 | 15.60 | 35.00 | 1.00 | 35.00 |
| 21-Dec | 20943.49 | 519.50 | 3856.23 | 981.52 | 3679.43 | 4264.94 | 15.06 | 36.00 | 1.00 | 36.00 |
| 22-Jan | 22420.54 | 630.15 | 3814.45 | 1244.60 | 3385.93 | 5263.40 | 15.79 | 37.00 | 1.00 | 37.00 |
| 22-Feb | 21580.96 | 586.81 | 4512.57 | 910.15 | 3209.39 | 3257.23 | 16.00 | 38.00 | 1.00 | 38.00 |
| 22-Mar | 28287.22 | 695.60 | 4239.88 | 1496.64 | 4998.46 | 6873.16 | 17.45 | 39.00 | 1.00 | 39.00 |
| 22-Apr | 28484.83 | 779.93 | 4407.86 | 1507.61 | 5157.66 | 6347.20 | 18.09 | 40.00 | 1.00 | 40.00 |
| 22-May | 23125.50 | 617.30 | 3742.85 | 1168.51 | 4041.12 | 4907.51 | 15.95 | 41.00 | 1.00 | 41.00 |
| 22-Jun | 26488.20 | 924.56 | 4093.41 | 966.75 | 4795.91 | 6206.68 | 16.39 | 42.00 | 1.00 | 42.00 |
| 22-Jul | 32558.61 | 786.03 | 4729.23 | 1918.02 | 4861.06 | 10141.03 | 18.09 | 43.00 | 1.00 | 43.00 |
| 22-Aug | 25832.69 | 671.25 | 3803.78 | 1353.65 | 4614.30 | 6081.25 | 14.79 | 44.00 | 1.00 | 44.00 |
| 22-Sep | 25568.46 | 472.94 | 3455.23 | 1235.95 | 5128.74 | 6654.72 | 12.29 | 45.00 | 1.00 | 45.00 |
| 22-Oct | 19149.30 | 545.19 | 3201.64 | 799.76 | 3862.21 | 3194.31 | 13.00 | 46.00 | 1.00 | 46.00 |
| 22-Nov | 25059.39 | 628.74 | 3833.39 | 1516.10 | 4977.09 | 4889.43 | 14.00 | 47.00 | 1.00 | 47.00 |
| 22-Dec | 28409.45 | 720.34 | 4675.32 | 1106.50 | 4883.87 | 6006.97 | 15.45 | 48.00 | 1.00 | 48.00 |
| 23-Jan | 10998.27 | 374.46 | 2380.80 | 401.75 | 1470.75 | 1758.92 | 9.00 | 49.00 | 1.00 | 49.00 |
| 23-Feb | 28650.83 | 684.93 | 4564.70 | 1497.48 | 3562.90 | 8238.95 | 15.67 | 50.00 | 1.00 | 50.00 |
| 23-Mar | 29219.73 | 755.25 | 4632.28 | 1627.09 | 3502.03 | 8686.86 | 15.49 | 51.00 | 1.00 | 51.00 |
| 23-Apr | 35674.19 | 685.35 | 5029.25 | 1887.92 | 4109.32 | 12543.19 | 15.59 | 52.00 | 1.00 | 52.00 |
| 23-May | 32522.14 | 872.21 | 6239.94 | 1425.90 | 3778.55 | 7567.70 | 15.45 | 53.00 | 1.00 | 53.00 |
| 23-Jun | 30041.33 | 713.23 | 4990.69 | 1800.23 | 3877.65 | 7135.73 | 15.52 | 54.00 | 1.00 | 54.00 |
| 23-Jul | 30921.61 | 964.48 | 4730.44 | 1862.06 | 5245.16 | 6032.14 | 15.46 | 55.00 | 1.00 | 55.00 |
| 23-Aug | 29468.23 | 788.64 | 4885.37 | 2002.26 | 3463.26 | 7069.47 | 18.26 | 56.00 | 1.00 | 56.00 |
| 23-Sep | 15530.25 | 416.05 | 3587.21 | 943.30 | 1331.14 | 2931.17 | 11.05 | 57.00 | 1.00 | 57.00 |
| 23-Oct | 20271.92 | 528.98 | 4373.57 | 1451.58 | 1823.59 | 3943.70 | 13.00 | 58.00 | 1.00 | 58.00 |
| 23-Nov | 29431.71 | 1180.05 | 4834.99 | 1748.36 | 3178.95 | 6082.33 | 16.94 | 59.00 | 1.00 | 59.00 |
| 23-Dec | 29127.36 | 1059.62 | 4867.09 | 1962.10 | 3021.14 | 7179.39 | 15.81 | 60.00 | 1.00 | 60.00 |
